# Supplementary material for: Lactate-mediated epigenetic reprogramming regulates formation of human pancreatic cancer-associated fibroblasts
Source: eLife. 2019 Nov 1;8:e50663. doi: 10.7554/eLife.50663 (PMC6874475; doi:10.7554/eLife.50663)
Supplement: Supplementary file 1. [file elife-50663-supp1.pptx]

## Slide 1
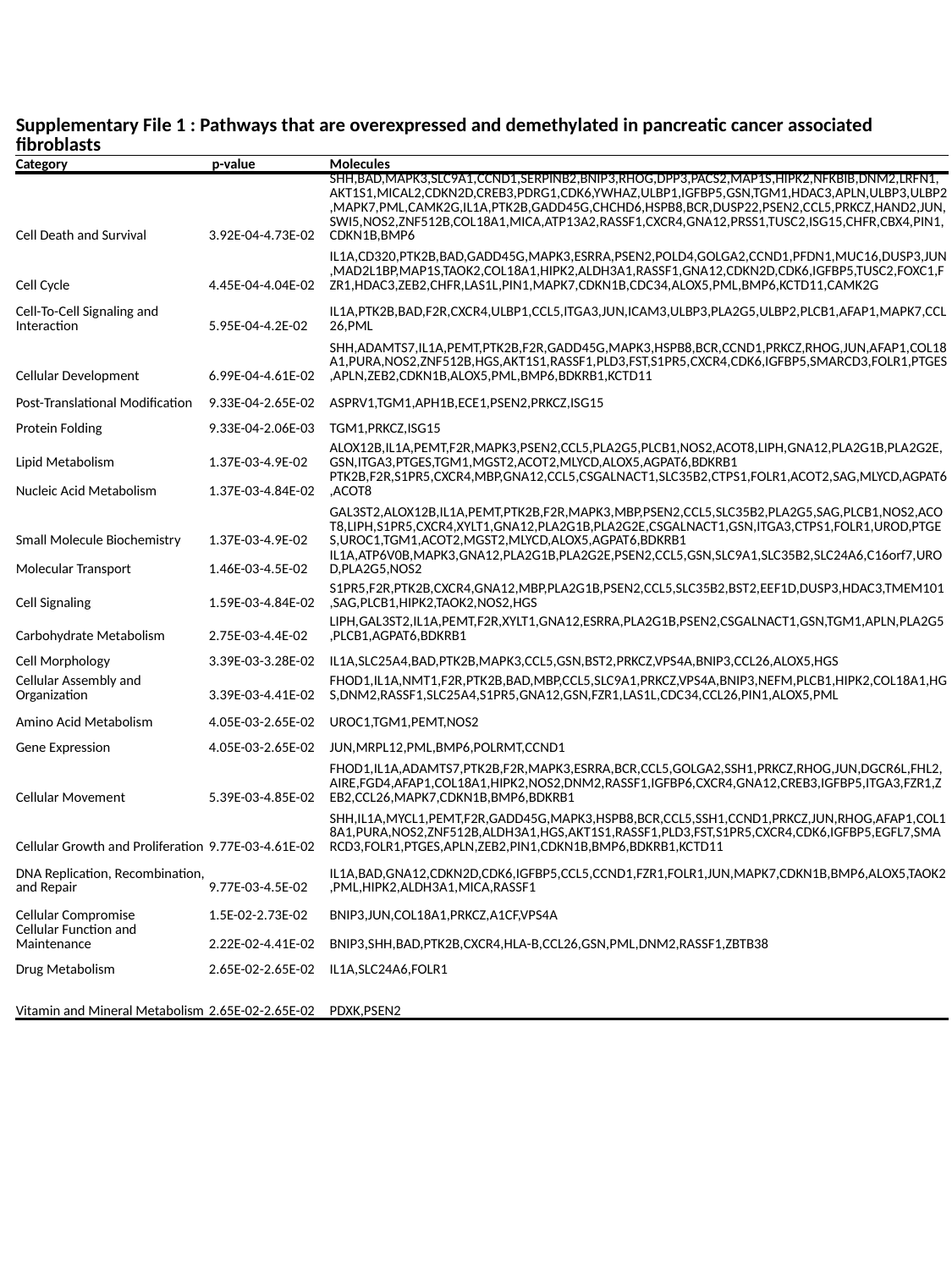

| Supplementary File 1 : Pathways that are overexpressed and demethylated in pancreatic cancer associated fibroblasts | | |
| --- | --- | --- |
| Category | p-value | Molecules |
| Cell Death and Survival | 3.92E-04-4.73E-02 | SHH,BAD,MAPK3,SLC9A1,CCND1,SERPINB2,BNIP3,RHOG,DPP3,PACS2,MAP1S,HIPK2,NFKBIB,DNM2,LRFN1,AKT1S1,MICAL2,CDKN2D,CREB3,PDRG1,CDK6,YWHAZ,ULBP1,IGFBP5,GSN,TGM1,HDAC3,APLN,ULBP3,ULBP2,MAPK7,PML,CAMK2G,IL1A,PTK2B,GADD45G,CHCHD6,HSPB8,BCR,DUSP22,PSEN2,CCL5,PRKCZ,HAND2,JUN,SWI5,NOS2,ZNF512B,COL18A1,MICA,ATP13A2,RASSF1,CXCR4,GNA12,PRSS1,TUSC2,ISG15,CHFR,CBX4,PIN1,CDKN1B,BMP6 |
| Cell Cycle | 4.45E-04-4.04E-02 | IL1A,CD320,PTK2B,BAD,GADD45G,MAPK3,ESRRA,PSEN2,POLD4,GOLGA2,CCND1,PFDN1,MUC16,DUSP3,JUN,MAD2L1BP,MAP1S,TAOK2,COL18A1,HIPK2,ALDH3A1,RASSF1,GNA12,CDKN2D,CDK6,IGFBP5,TUSC2,FOXC1,FZR1,HDAC3,ZEB2,CHFR,LAS1L,PIN1,MAPK7,CDKN1B,CDC34,ALOX5,PML,BMP6,KCTD11,CAMK2G |
| Cell-To-Cell Signaling and Interaction | 5.95E-04-4.2E-02 | IL1A,PTK2B,BAD,F2R,CXCR4,ULBP1,CCL5,ITGA3,JUN,ICAM3,ULBP3,PLA2G5,ULBP2,PLCB1,AFAP1,MAPK7,CCL26,PML |
| Cellular Development | 6.99E-04-4.61E-02 | SHH,ADAMTS7,IL1A,PEMT,PTK2B,F2R,GADD45G,MAPK3,HSPB8,BCR,CCND1,PRKCZ,RHOG,JUN,AFAP1,COL18A1,PURA,NOS2,ZNF512B,HGS,AKT1S1,RASSF1,PLD3,FST,S1PR5,CXCR4,CDK6,IGFBP5,SMARCD3,FOLR1,PTGES,APLN,ZEB2,CDKN1B,ALOX5,PML,BMP6,BDKRB1,KCTD11 |
| Post-Translational Modification | 9.33E-04-2.65E-02 | ASPRV1,TGM1,APH1B,ECE1,PSEN2,PRKCZ,ISG15 |
| Protein Folding | 9.33E-04-2.06E-03 | TGM1,PRKCZ,ISG15 |
| Lipid Metabolism | 1.37E-03-4.9E-02 | ALOX12B,IL1A,PEMT,F2R,MAPK3,PSEN2,CCL5,PLA2G5,PLCB1,NOS2,ACOT8,LIPH,GNA12,PLA2G1B,PLA2G2E,GSN,ITGA3,PTGES,TGM1,MGST2,ACOT2,MLYCD,ALOX5,AGPAT6,BDKRB1 |
| Nucleic Acid Metabolism | 1.37E-03-4.84E-02 | PTK2B,F2R,S1PR5,CXCR4,MBP,GNA12,CCL5,CSGALNACT1,SLC35B2,CTPS1,FOLR1,ACOT2,SAG,MLYCD,AGPAT6,ACOT8 |
| Small Molecule Biochemistry | 1.37E-03-4.9E-02 | GAL3ST2,ALOX12B,IL1A,PEMT,PTK2B,F2R,MAPK3,MBP,PSEN2,CCL5,SLC35B2,PLA2G5,SAG,PLCB1,NOS2,ACOT8,LIPH,S1PR5,CXCR4,XYLT1,GNA12,PLA2G1B,PLA2G2E,CSGALNACT1,GSN,ITGA3,CTPS1,FOLR1,UROD,PTGES,UROC1,TGM1,ACOT2,MGST2,MLYCD,ALOX5,AGPAT6,BDKRB1 |
| Molecular Transport | 1.46E-03-4.5E-02 | IL1A,ATP6V0B,MAPK3,GNA12,PLA2G1B,PLA2G2E,PSEN2,CCL5,GSN,SLC9A1,SLC35B2,SLC24A6,C16orf7,UROD,PLA2G5,NOS2 |
| Cell Signaling | 1.59E-03-4.84E-02 | S1PR5,F2R,PTK2B,CXCR4,GNA12,MBP,PLA2G1B,PSEN2,CCL5,SLC35B2,BST2,EEF1D,DUSP3,HDAC3,TMEM101,SAG,PLCB1,HIPK2,TAOK2,NOS2,HGS |
| Carbohydrate Metabolism | 2.75E-03-4.4E-02 | LIPH,GAL3ST2,IL1A,PEMT,F2R,XYLT1,GNA12,ESRRA,PLA2G1B,PSEN2,CSGALNACT1,GSN,TGM1,APLN,PLA2G5,PLCB1,AGPAT6,BDKRB1 |
| Cell Morphology | 3.39E-03-3.28E-02 | IL1A,SLC25A4,BAD,PTK2B,MAPK3,CCL5,GSN,BST2,PRKCZ,VPS4A,BNIP3,CCL26,ALOX5,HGS |
| Cellular Assembly and Organization | 3.39E-03-4.41E-02 | FHOD1,IL1A,NMT1,F2R,PTK2B,BAD,MBP,CCL5,SLC9A1,PRKCZ,VPS4A,BNIP3,NEFM,PLCB1,HIPK2,COL18A1,HGS,DNM2,RASSF1,SLC25A4,S1PR5,GNA12,GSN,FZR1,LAS1L,CDC34,CCL26,PIN1,ALOX5,PML |
| Amino Acid Metabolism | 4.05E-03-2.65E-02 | UROC1,TGM1,PEMT,NOS2 |
| Gene Expression | 4.05E-03-2.65E-02 | JUN,MRPL12,PML,BMP6,POLRMT,CCND1 |
| Cellular Movement | 5.39E-03-4.85E-02 | FHOD1,IL1A,ADAMTS7,PTK2B,F2R,MAPK3,ESRRA,BCR,CCL5,GOLGA2,SSH1,PRKCZ,RHOG,JUN,DGCR6L,FHL2,AIRE,FGD4,AFAP1,COL18A1,HIPK2,NOS2,DNM2,RASSF1,IGFBP6,CXCR4,GNA12,CREB3,IGFBP5,ITGA3,FZR1,ZEB2,CCL26,MAPK7,CDKN1B,BMP6,BDKRB1 |
| Cellular Growth and Proliferation | 9.77E-03-4.61E-02 | SHH,IL1A,MYCL1,PEMT,F2R,GADD45G,MAPK3,HSPB8,BCR,CCL5,SSH1,CCND1,PRKCZ,JUN,RHOG,AFAP1,COL18A1,PURA,NOS2,ZNF512B,ALDH3A1,HGS,AKT1S1,RASSF1,PLD3,FST,S1PR5,CXCR4,CDK6,IGFBP5,EGFL7,SMARCD3,FOLR1,PTGES,APLN,ZEB2,PIN1,CDKN1B,BMP6,BDKRB1,KCTD11 |
| DNA Replication, Recombination, and Repair | 9.77E-03-4.5E-02 | IL1A,BAD,GNA12,CDKN2D,CDK6,IGFBP5,CCL5,CCND1,FZR1,FOLR1,JUN,MAPK7,CDKN1B,BMP6,ALOX5,TAOK2,PML,HIPK2,ALDH3A1,MICA,RASSF1 |
| Cellular Compromise | 1.5E-02-2.73E-02 | BNIP3,JUN,COL18A1,PRKCZ,A1CF,VPS4A |
| Cellular Function and Maintenance | 2.22E-02-4.41E-02 | BNIP3,SHH,BAD,PTK2B,CXCR4,HLA-B,CCL26,GSN,PML,DNM2,RASSF1,ZBTB38 |
| Drug Metabolism | 2.65E-02-2.65E-02 | IL1A,SLC24A6,FOLR1 |
| Vitamin and Mineral Metabolism | 2.65E-02-2.65E-02 | PDXK,PSEN2 |
