# Supplementary material for: Lactate-mediated epigenetic reprogramming regulates formation of human pancreatic cancer-associated fibroblasts
Source: eLife. 2019 Nov 1;8:e50663. doi: 10.7554/eLife.50663 (PMC6874475; doi:10.7554/eLife.50663)
Supplement: Supplementary file 2. [file elife-50663-supp2.pptx]

## Slide 1
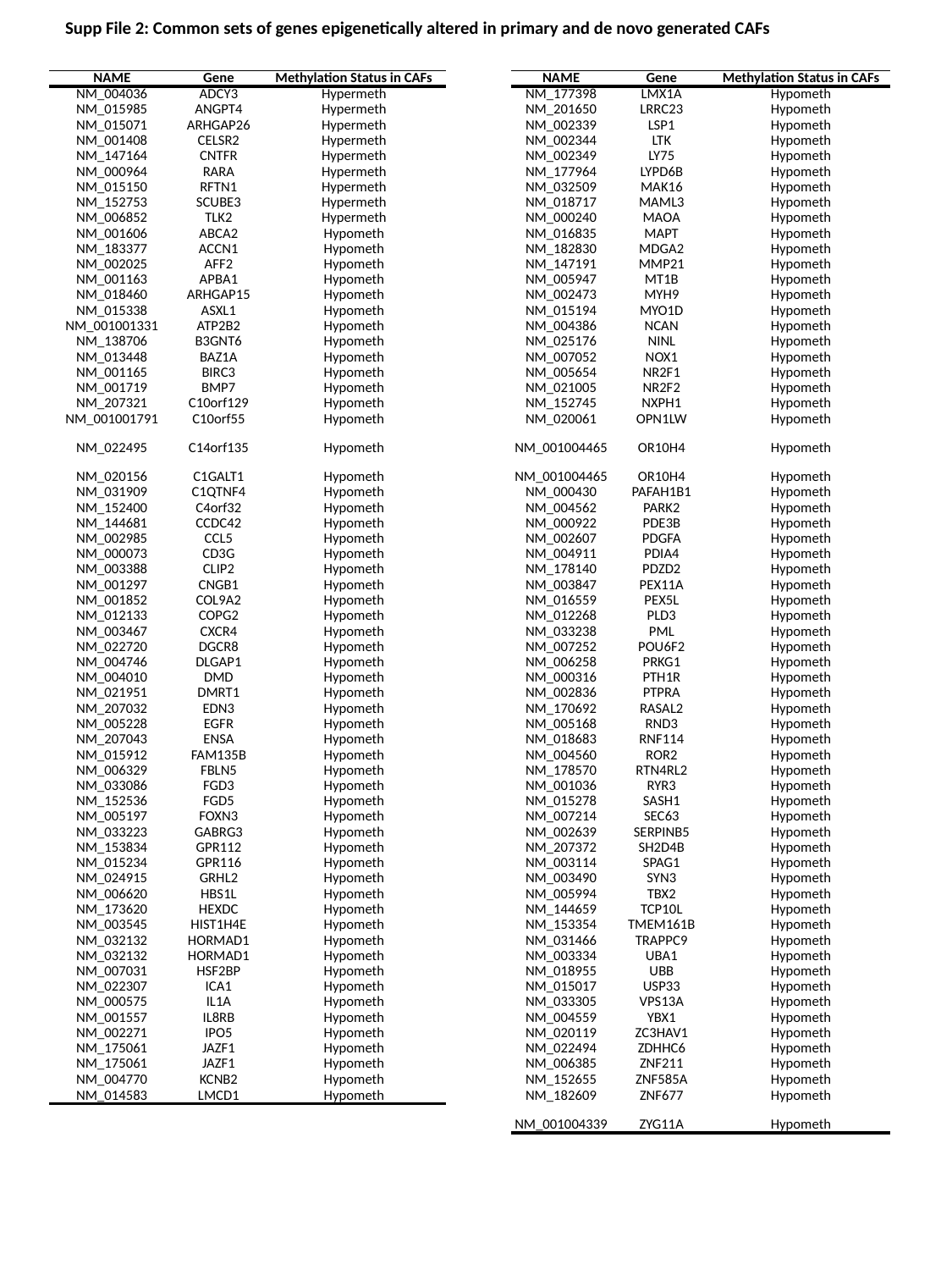

Supp File 2: Common sets of genes epigenetically altered in primary and de novo generated CAFs
| NAME | Gene | Methylation Status in CAFs | | NAME | Gene | Methylation Status in CAFs |
| --- | --- | --- | --- | --- | --- | --- |
| NM\_004036 | ADCY3 | Hypermeth | | NM\_177398 | LMX1A | Hypometh |
| NM\_015985 | ANGPT4 | Hypermeth | | NM\_201650 | LRRC23 | Hypometh |
| NM\_015071 | ARHGAP26 | Hypermeth | | NM\_002339 | LSP1 | Hypometh |
| NM\_001408 | CELSR2 | Hypermeth | | NM\_002344 | LTK | Hypometh |
| NM\_147164 | CNTFR | Hypermeth | | NM\_002349 | LY75 | Hypometh |
| NM\_000964 | RARA | Hypermeth | | NM\_177964 | LYPD6B | Hypometh |
| NM\_015150 | RFTN1 | Hypermeth | | NM\_032509 | MAK16 | Hypometh |
| NM\_152753 | SCUBE3 | Hypermeth | | NM\_018717 | MAML3 | Hypometh |
| NM\_006852 | TLK2 | Hypermeth | | NM\_000240 | MAOA | Hypometh |
| NM\_001606 | ABCA2 | Hypometh | | NM\_016835 | MAPT | Hypometh |
| NM\_183377 | ACCN1 | Hypometh | | NM\_182830 | MDGA2 | Hypometh |
| NM\_002025 | AFF2 | Hypometh | | NM\_147191 | MMP21 | Hypometh |
| NM\_001163 | APBA1 | Hypometh | | NM\_005947 | MT1B | Hypometh |
| NM\_018460 | ARHGAP15 | Hypometh | | NM\_002473 | MYH9 | Hypometh |
| NM\_015338 | ASXL1 | Hypometh | | NM\_015194 | MYO1D | Hypometh |
| NM\_001001331 | ATP2B2 | Hypometh | | NM\_004386 | NCAN | Hypometh |
| NM\_138706 | B3GNT6 | Hypometh | | NM\_025176 | NINL | Hypometh |
| NM\_013448 | BAZ1A | Hypometh | | NM\_007052 | NOX1 | Hypometh |
| NM\_001165 | BIRC3 | Hypometh | | NM\_005654 | NR2F1 | Hypometh |
| NM\_001719 | BMP7 | Hypometh | | NM\_021005 | NR2F2 | Hypometh |
| NM\_207321 | C10orf129 | Hypometh | | NM\_152745 | NXPH1 | Hypometh |
| NM\_001001791 | C10orf55 | Hypometh | | NM\_020061 | OPN1LW | Hypometh |
| NM\_022495 | C14orf135 | Hypometh | | NM\_001004465 | OR10H4 | Hypometh |
| NM\_020156 | C1GALT1 | Hypometh | | NM\_001004465 | OR10H4 | Hypometh |
| NM\_031909 | C1QTNF4 | Hypometh | | NM\_000430 | PAFAH1B1 | Hypometh |
| NM\_152400 | C4orf32 | Hypometh | | NM\_004562 | PARK2 | Hypometh |
| NM\_144681 | CCDC42 | Hypometh | | NM\_000922 | PDE3B | Hypometh |
| NM\_002985 | CCL5 | Hypometh | | NM\_002607 | PDGFA | Hypometh |
| NM\_000073 | CD3G | Hypometh | | NM\_004911 | PDIA4 | Hypometh |
| NM\_003388 | CLIP2 | Hypometh | | NM\_178140 | PDZD2 | Hypometh |
| NM\_001297 | CNGB1 | Hypometh | | NM\_003847 | PEX11A | Hypometh |
| NM\_001852 | COL9A2 | Hypometh | | NM\_016559 | PEX5L | Hypometh |
| NM\_012133 | COPG2 | Hypometh | | NM\_012268 | PLD3 | Hypometh |
| NM\_003467 | CXCR4 | Hypometh | | NM\_033238 | PML | Hypometh |
| NM\_022720 | DGCR8 | Hypometh | | NM\_007252 | POU6F2 | Hypometh |
| NM\_004746 | DLGAP1 | Hypometh | | NM\_006258 | PRKG1 | Hypometh |
| NM\_004010 | DMD | Hypometh | | NM\_000316 | PTH1R | Hypometh |
| NM\_021951 | DMRT1 | Hypometh | | NM\_002836 | PTPRA | Hypometh |
| NM\_207032 | EDN3 | Hypometh | | NM\_170692 | RASAL2 | Hypometh |
| NM\_005228 | EGFR | Hypometh | | NM\_005168 | RND3 | Hypometh |
| NM\_207043 | ENSA | Hypometh | | NM\_018683 | RNF114 | Hypometh |
| NM\_015912 | FAM135B | Hypometh | | NM\_004560 | ROR2 | Hypometh |
| NM\_006329 | FBLN5 | Hypometh | | NM\_178570 | RTN4RL2 | Hypometh |
| NM\_033086 | FGD3 | Hypometh | | NM\_001036 | RYR3 | Hypometh |
| NM\_152536 | FGD5 | Hypometh | | NM\_015278 | SASH1 | Hypometh |
| NM\_005197 | FOXN3 | Hypometh | | NM\_007214 | SEC63 | Hypometh |
| NM\_033223 | GABRG3 | Hypometh | | NM\_002639 | SERPINB5 | Hypometh |
| NM\_153834 | GPR112 | Hypometh | | NM\_207372 | SH2D4B | Hypometh |
| NM\_015234 | GPR116 | Hypometh | | NM\_003114 | SPAG1 | Hypometh |
| NM\_024915 | GRHL2 | Hypometh | | NM\_003490 | SYN3 | Hypometh |
| NM\_006620 | HBS1L | Hypometh | | NM\_005994 | TBX2 | Hypometh |
| NM\_173620 | HEXDC | Hypometh | | NM\_144659 | TCP10L | Hypometh |
| NM\_003545 | HIST1H4E | Hypometh | | NM\_153354 | TMEM161B | Hypometh |
| NM\_032132 | HORMAD1 | Hypometh | | NM\_031466 | TRAPPC9 | Hypometh |
| NM\_032132 | HORMAD1 | Hypometh | | NM\_003334 | UBA1 | Hypometh |
| NM\_007031 | HSF2BP | Hypometh | | NM\_018955 | UBB | Hypometh |
| NM\_022307 | ICA1 | Hypometh | | NM\_015017 | USP33 | Hypometh |
| NM\_000575 | IL1A | Hypometh | | NM\_033305 | VPS13A | Hypometh |
| NM\_001557 | IL8RB | Hypometh | | NM\_004559 | YBX1 | Hypometh |
| NM\_002271 | IPO5 | Hypometh | | NM\_020119 | ZC3HAV1 | Hypometh |
| NM\_175061 | JAZF1 | Hypometh | | NM\_022494 | ZDHHC6 | Hypometh |
| NM\_175061 | JAZF1 | Hypometh | | NM\_006385 | ZNF211 | Hypometh |
| NM\_004770 | KCNB2 | Hypometh | | NM\_152655 | ZNF585A | Hypometh |
| NM\_014583 | LMCD1 | Hypometh | | NM\_182609 | ZNF677 | Hypometh |
| | | | | NM\_001004339 | ZYG11A | Hypometh |
| | | | | | | |
