# Supplementary material for: Lactate-mediated epigenetic reprogramming regulates formation of human pancreatic cancer-associated fibroblasts
Source: eLife. 2019 Nov 1;8:e50663. doi: 10.7554/eLife.50663 (PMC6874475; doi:10.7554/eLife.50663)
Supplement: Supplementary file 3. [file elife-50663-supp3.pptx]

## Slide 1
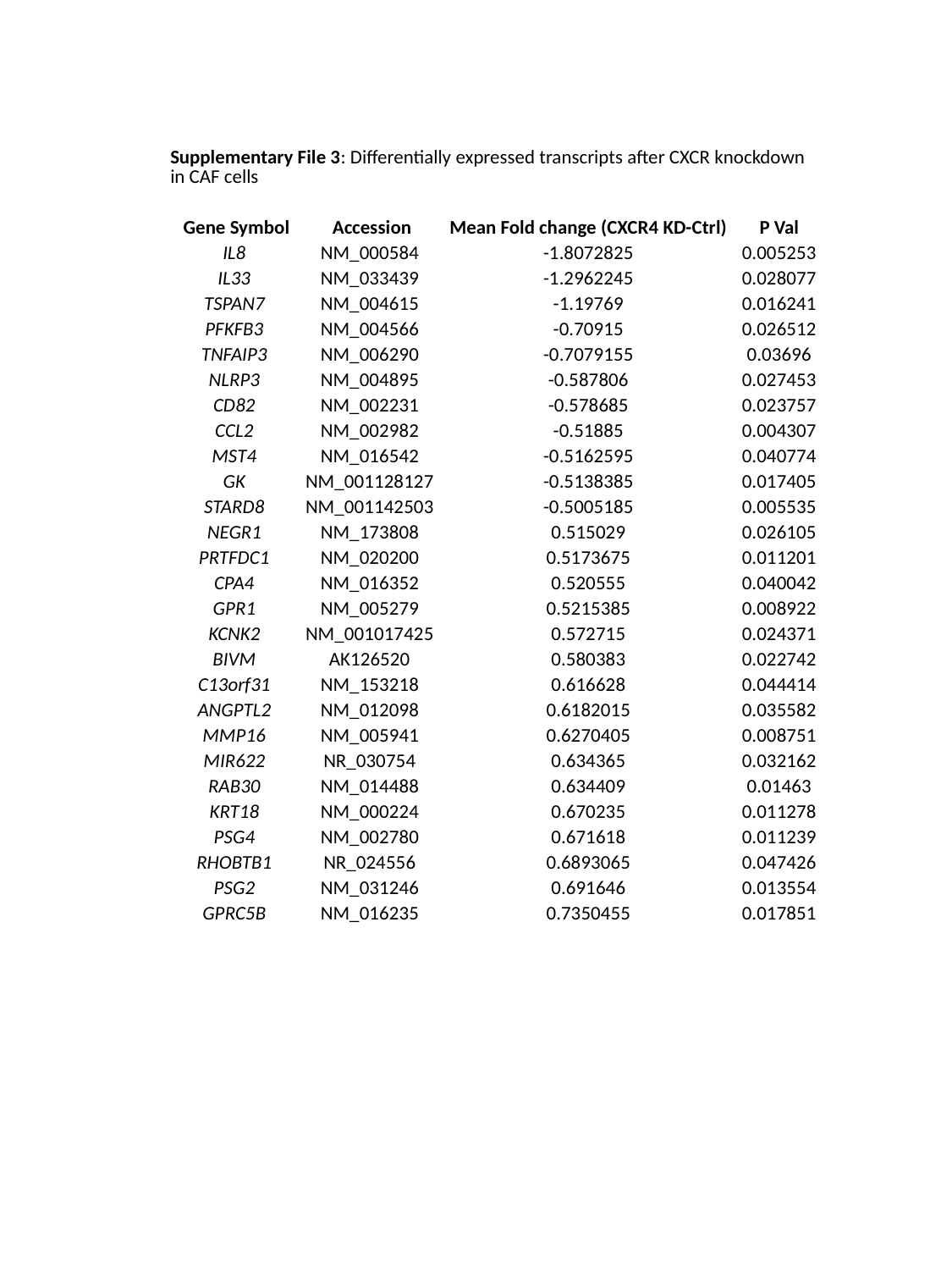

| Supplementary File 3: Differentially expressed transcripts after CXCR knockdown in CAF cells | | | |
| --- | --- | --- | --- |
| | | | |
| Gene Symbol | Accession | Mean Fold change (CXCR4 KD-Ctrl) | P Val |
| IL8 | NM\_000584 | -1.8072825 | 0.005253 |
| IL33 | NM\_033439 | -1.2962245 | 0.028077 |
| TSPAN7 | NM\_004615 | -1.19769 | 0.016241 |
| PFKFB3 | NM\_004566 | -0.70915 | 0.026512 |
| TNFAIP3 | NM\_006290 | -0.7079155 | 0.03696 |
| NLRP3 | NM\_004895 | -0.587806 | 0.027453 |
| CD82 | NM\_002231 | -0.578685 | 0.023757 |
| CCL2 | NM\_002982 | -0.51885 | 0.004307 |
| MST4 | NM\_016542 | -0.5162595 | 0.040774 |
| GK | NM\_001128127 | -0.5138385 | 0.017405 |
| STARD8 | NM\_001142503 | -0.5005185 | 0.005535 |
| NEGR1 | NM\_173808 | 0.515029 | 0.026105 |
| PRTFDC1 | NM\_020200 | 0.5173675 | 0.011201 |
| CPA4 | NM\_016352 | 0.520555 | 0.040042 |
| GPR1 | NM\_005279 | 0.5215385 | 0.008922 |
| KCNK2 | NM\_001017425 | 0.572715 | 0.024371 |
| BIVM | AK126520 | 0.580383 | 0.022742 |
| C13orf31 | NM\_153218 | 0.616628 | 0.044414 |
| ANGPTL2 | NM\_012098 | 0.6182015 | 0.035582 |
| MMP16 | NM\_005941 | 0.6270405 | 0.008751 |
| MIR622 | NR\_030754 | 0.634365 | 0.032162 |
| RAB30 | NM\_014488 | 0.634409 | 0.01463 |
| KRT18 | NM\_000224 | 0.670235 | 0.011278 |
| PSG4 | NM\_002780 | 0.671618 | 0.011239 |
| RHOBTB1 | NR\_024556 | 0.6893065 | 0.047426 |
| PSG2 | NM\_031246 | 0.691646 | 0.013554 |
| GPRC5B | NM\_016235 | 0.7350455 | 0.017851 |
